# Morse potential specific heat with applications: an integral equations theory based

**DOI:** 10.1186/s13065-022-00811-3

**Published:** 2022-03-27

**Authors:** Marwan Al-Raeei

**Affiliations:** Faculty of Sciences, Damascus University, Damascus, Syrian Arab Republic

**Keywords:** Specific heat, Morse potential, Quantum oscillator, Fractional volume, Integral equations

## Abstract

The specific heat in its molar form or mass form is a significant thermal property in the study of the thermal capacity of the described system. There are two basic methods for the determination of the molar specific heat capacity, one of them is the experimental procedure and the other is the theoretical procedure. The present study deals with finding a formula of the molar specific heat capacity using the theory of the integral equations for Morse interaction which is a very important potential for the study of the general oscillations in the quantum mechanics. We use the approximation (Mean-Spherical) for finding the total energy of the compositions described by Morse interaction. We find two formulas of the heat capacity, one at a constant pressure and the other at a constant volume. We conclude that the Morse molar specific heat is temperature dependent via the inverse square low with respect to temperature. Besides, we find that the Morse molar specific heat is proportional to the square of the Morse interaction well depth. Also, we find that the Morse molar specific heat depends on the particles’ diameter, the bond distance of Morse interaction, the width parameter of Morse interaction, and the volumetric density of the system. We apply the formula of the specific heat for finding the specific heat of the vibrational part for two dimer which are the lithium and caesium dimers and for the hydrogen fluoride, hydrogen chloride, nitrogen, and hydrogen molecules.

## Introduction

One of the most significant thermal properties is the molar specific-heat, which is considered the main properties in the calorimeters measurements. The latter measurements is the basic experimental procedures for finding the values of the molar specific-heat for a specific system, and also, the molar specific-heat capacity can be determined through a theoretical procedures derived from the principles of the thermodynamics or derived from the principles of the statistical mechanics which we focus on. We employ the theory of the closed integral equations of the statistical-mechanics for finding a formula of the full energy of a system described by a Morse interaction and from this formula, we find a formula for the molar specific-heat capacity of the Morse interaction. The volume-heat capacity is found from the equation:

1 $$ C^{V} (T) = {\rm E}_{T} $$

The right-side of the previous equation represents the first derivative of the energy with respect to the absolute temperature. We use the previous main equation for finding the Molar specific-heat for the Morse potential which is an important potential for describing the vibrational cases, especially, in quantum mechanics [1]. In present work, we employ the integral equations theory solutions for the low density systems for deriving an equation of the molar specific-heat capacity for the Morse interaction where the theory of integral equations has multiple applications in lots of properties of the physical systems. For instance, the theory was applied for discussing: some polymeric systems with experimental data [2], some diatomic molecules [3], the nematic case [4], some electrical properties in the liquids [5], the hard sphere as an analytical study [6], deriving equations of state for a specific type of dispersion [7], multi-atomic systems of the fluids [8], the structure-factor of a charged system [9]. In this study, we apply the mean-spherical-approximation for deriving a specific heat formula of Morse potential which can be derived in multiple form such as:

2 $$ U_{Morse} (r) = {\rm K}_{0} \left[ {\exp \left( {2a\left( {r_{0} - r} \right) - 2\exp \left( {a\left( {r_{0} - r} \right)} \right)} \right)} \right] $$

where Κ_0_ represents the energy (equilibrium) of the interaction potential, *a* is the width of well parameter and *r*_*0*_ is the equilibrium bond distance. The Morse potential has multiple applications in lots of chemical physics subjects, for instance: the discussing of the thermal properties of a specific system such as the diamond class materials and finding the constants of the vibrational force and the elastic properties [10–12], the discussing of the correlations in alloys phases [13], the discussing of the spectral analysis [14], in the study of some quantum effects [15, 16], discussing the energy vibrational states [17, 18], discussing the alpha decay [19], the study of the structures with other potentials [20–22], the study of the some dimers where this potential has wide applications [22, 23]. In the section-2 of this article, we illustrate the method of deriving of the specific heat equation, and in the section-3, we discussed some aspects of the equation which we derive in addition to the applications of it. While in the last section of the article, we inserted some conclusions points.

## The heat volumetric capacity for Morse potential

One of the most significant equations in the integral equation theory is the Ornstein_Zernike (O_Z) equation which describes the correlation between the particles in the system as direct correlation and indirect correlation and this equation is given as follows:

3 $$ h^{t} (r) \equiv c^{**} (r) + c^{*} (r) $$

where *r* is the distance of the particles, *n* is number of the density, c^*^(r) is the correlation function-direct while *h*^*t*^*(r)* is the total correlation function and c^**^(r) is the correlation function-indirect which is:

4 $$ c^{**} (r) = n\int {c^{*} \left( {\left| {\vec{r} - \vec{r}^{\prime}} \right|} \right)} h^{t} \left( {r^{\prime}} \right)d\vec{r}^{\prime} $$

As we see from the two equations-3 and 4, the solutions of the Ornstein_Zernike equation can be found if we employ another equation which can be resulted from many approximations formulas included in the simple fluids theory such as the mean spherical approximation MSA and other approximations. In this work, we use MS-approximation for deriving the molar specific-heat capacity of the Morse potential. We start from the general relationship of the full energy:

5 $$ {\rm E} = \frac{4n\pi N}{2}\int_{full} {r^{2} g(r)U_{Morse} (r)} dr + \frac{{3Tk_{B} N}}{2} $$

*N* represent a number of particles in the described system and the constant in the last term of the equation is Boltzmann constant. Now, if we use the MS-approximation and the formula of the Morse potential in the full energy formula, we find:

6 $$ {\rm E} = \frac{{3Tk_{B} N}}{2} + G_{1} + \frac{{4n\pi {\rm K}_{0} N}}{2}G_{2} $$

where:

7 $$ G_{1} = \int\limits_{0}^{d} {r^{2} drg_{0} (r)U_{Morse} (r)} $$

8 $$ G_{2} = \int\limits_{d}^{\infty } {\left[ {\exp \left( {2a\left( {r_{0} - r} \right)} \right) - 2\exp \left( {a\left( {r_{0} - r} \right)} \right)} \right]\left[ {r^{2} - \frac{{{\rm K}_{0} }}{{Tk_{B} }}r^{2} \exp \left( {2a\left( {r_{0} - r} \right)} \right) + \,2\frac{{{\rm K}_{0} }}{{Tk_{B} }}r\exp \left( {a\left( {r_{0} - r} \right)} \right)} \right]dr} $$

Or in another form:

9 $$ {\rm E} = \frac{{3Tk_{B} N}}{2} + \frac{{4n\pi {\rm K}_{0} N}}{2}G_{3} + G_{4} $$

where:

10 $$ G_{3} = \int\limits_{d}^{\infty } {\left[ {\left( {r^{2} - 4\frac{{D_{0} }}{{Tk_{B} }}r^{2} } \right)\exp \left( {2a\left( {r_{0} - r} \right)} \right)} \right]dr} $$

11 $$ G_{4} = \int\limits_{d}^{\infty } {\left[ {\frac{{{\rm K}_{0} }}{{Tk_{B} }}r^{2} \exp \left( {4a\left( {r_{0} - r} \right)} \right) + \,4\frac{{{\rm K}_{0} }}{{Tk_{B} }}r^{2} \exp \left( {3a\left( {r_{0} - r} \right)} \right) - \,2r^{2} \exp \left( {a\left( {r_{0} - r} \right)} \right)} \right]dr} $$

Or in simpler form:

12 $$ {\rm E} = \frac{{3Tk_{B} N}}{2} + \frac{{4n\pi {\rm K}_{0} N}}{2}\left[ {\left( {1 - 4\frac{{{\rm K}_{0} }}{{Tk_{B} }}} \right){\rm I}_{2} + 4\frac{{{\rm K}_{0} }}{{Tk_{B} }}{\rm I}_{3} - 2{\rm I}_{1} - \frac{{{\rm K}_{0} }}{{Tk_{B} }}{\rm I}_{4} } \right] $$

where:

13 $$ {\text{I}}_{1} = \eta_{1} \left( {r_{0} ,a} \right)\int\limits_{d}^{\infty } {\mathop y\nolimits^{*2} } dy^{*} \exp \left( { - y^{*} } \right) $$

14 $$ {\text{I}}_{2} = \eta_{2} \left( {r_{0} ,a} \right)\int\limits_{d}^{\infty } {\mathop y\nolimits^{*2} } dy^{*} \exp \left( { - 2y^{*} } \right) $$

15 $$ {\text{I}}_{3} = \eta_{3} \left( {a,r_{0} } \right)\int\limits_{d}^{\infty } {\mathop y\nolimits^{*2} } dy^{*} \exp \left( { - 3y^{*} } \right) $$

16 $$ {\text{I}}_{4} = C_{2} \left( {a,r_{0} } \right)\int\limits_{d}^{\infty } {\mathop y\nolimits^{*4} } dy^{*} \exp \left( { - 4y^{*} } \right) $$

And we used the following reduced Morse interaction parameters:

17 $$ ay^{**} = y^{*} $$

18 $$ r = y^{**} + r_{0} $$

19 $$ y^{**} a = y^{***} $$

The integral by partition method can be applied to the integrals in the four Eqs. (13–16) and using the reduced parameters we find that the full energy of the system written as follows:

20 $$ {\rm E} = \frac{{3Tk_{B} N}}{2} + \frac{{4n\pi {\rm K}_{0} N}}{2}\left[ \begin{gathered} \frac{1}{2}\left( {1 - 4\frac{{{\rm K}_{0} }}{{Tk_{B} }}} \right)\exp \left( {2a\left( {r_{0} - d} \right)} \right)\left( {\frac{{d^{2} }}{a} + \frac{d}{{a^{2} }} + \frac{1}{{2a^{3} }}} \right) \hfill \\ +\,4\frac{{{\rm K}_{0} }}{{3Tk_{B} }}\exp \left( {3a\left( {r_{0} - d} \right)} \right)\left( {\frac{{d^{2} }}{a} + \frac{2d}{{3a^{2} }} + \frac{2}{{9a^{3} }}} \right)  \hfill \\ -\,2\exp \left( {a\left( {r_{0} - d} \right)} \right)\left( {\frac{{d^{2} }}{a} + \frac{2d}{{a^{2} }} + \frac{2}{{a^{3} }}} \right) - \frac{{{\rm K}_{0} }}{{4Tk_{B} }}\exp \left( {4a\left( {r_{0} - d} \right)} \right)\left( {\frac{{d^{2} }}{a} + \frac{d}{{2a^{2} }} + \frac{1}{{8a^{3} }}} \right) \hfill \\ \end{gathered} \right] $$

If we apply the first equation in the work on the equation-20, we find that the heat volumetric capacity is given as:

21 $$ C^{V} (T) = \frac{{3k_{B} N}}{2} + \frac{{2n\pi {\rm K}_{0}^{2} N}}{{k_{B} T^{2} }}\left[ \begin{gathered} 2\exp \left( {2a\left( {r_{0} - d} \right)} \right)\left( {\frac{{d^{2} }}{a} + \frac{d}{{a^{2} }} + \frac{1}{{2a^{3} }}} \right) \hfill \\ + \frac{{\exp \left( {4a\left( {r_{0} - d} \right)} \right)}}{4}\left( {\frac{{d^{2} }}{a} + \frac{d}{{2a^{2} }} + \frac{1}{{8a^{3} }}} \right) - \frac{4}{3}\exp \left( {3a\left( {r_{0} - d} \right)} \right)\left( {\frac{{d^{2} }}{a} + \frac{2d}{{3a^{2} }} + \frac{2}{{9a^{3} }}} \right) \hfill \\ \end{gathered} \right] $$

## Results and discussion

### The Morse and the kinetic parts of the specific heat

The formula (21) is the main relationship which we found in the present work which represent the heat volumetric capacity. First, we can find the heat capacity at constant pressure from the relationship which relates with the heat volumetric capacity as follows:

22 $$ C^{p} (T) - C^{V} (T) = V\chi (T)\alpha^{2} T $$

Which gives us:

23 $$ C^{p} (T) = \,\frac{{3k_{B} N}}{2} + \frac{{2n\pi {\rm K}_{0}^{2} N}}{{k_{B} T^{2} }}\left[ \begin{gathered} 2\exp \left( {2a\left( {r_{0} - d} \right)} \right)\left( {\frac{{d^{2} }}{a} + \frac{d}{{a^{2} }} + \frac{1}{{2a^{3} }}} \right) \hfill \\ + \frac{{\exp \left( {4a\left( {r_{0} - d} \right)} \right)}}{4}\left( {\frac{{d^{2} }}{a} + \frac{d}{{2a^{2} }} + \frac{1}{{8a^{3} }}} \right) - \frac{4}{3}\exp \left( {3a\left( {r_{0} - d} \right)} \right)\left( {\frac{{d^{2} }}{a} + \frac{2d}{{3a^{2} }} + \frac{2}{{9a^{3} }}} \right) \hfill \\ \end{gathered} \right] + V\alpha^{2} \chi (T)T $$

where *χ(Τ)* is the thermal-compressibility, while *α* is the thermal expansion coefficient of the described system. However, we have two formulas of the heat capacity (pressure and volume), the two are semi-equal for the compositions which had a very small value of the thermal expansion parameter, for instance, soft materials.

Besides, we find the molar formalism of the volumetric heat capacity as follows:

24 $$ C^{V} (T) = \frac{3R}{2} + \frac{{2n\pi R{\rm K}_{0}^{2} }}{{k_{B}^{2} T^{2} }}\left[ \begin{gathered} 2\exp \left( {2a\left( {r_{0} - d} \right)} \right)\left( {\frac{{d^{2} }}{a} + \frac{d}{{a^{2} }} + \frac{1}{{2a^{3} }}} \right) \hfill \\ + \frac{{\exp \left( {4a\left( {r_{0} - d} \right)} \right)}}{4}\left( {\frac{{d^{2} }}{a} + \frac{d}{{2a^{2} }} + \frac{1}{{8a^{3} }}} \right) - \frac{4}{3}\exp \left( {3a\left( {r_{0} - d} \right)} \right)\left( {\frac{{d^{2} }}{a} + \frac{2d}{{3a^{2} }} + \frac{2}{{9a^{3} }}} \right) \hfill \\ \end{gathered} \right] $$

Which represents the specific heat for a one mole of the described composition. As we see from the equation-24, the molar specific heat can be written as:

25 $$ C^{V} (T) = C_{Id}^{V} (T) + C_{M}^{V} (T) $$

With:

26 $$ C_{Id}^{V} (T) \equiv \frac{3R}{2} $$

27 $$ C_{M}^{V} (T) \equiv \frac{{2n\pi R{\rm K}_{0}^{2} }}{{k_{B}^{2} T^{2} }}\left[ \begin{gathered} 2\exp \left( {2a\left( {r_{0} - d} \right)} \right)\left( {\frac{{d^{2} }}{a} + \frac{d}{{a^{2} }} + \frac{1}{{2a^{3} }}} \right) \hfill \\ + \frac{{\exp \left( {4a\left( {r_{0} - d} \right)} \right)}}{4}\left( {\frac{{d^{2} }}{a} + \frac{d}{{2a^{2} }} + \frac{1}{{8a^{3} }}} \right) - \frac{4}{3}\exp \left( {3a\left( {r_{0} - d} \right)} \right)\left( {\frac{{d^{2} }}{a} + \frac{2d}{{3a^{2} }} + \frac{2}{{9a^{3} }}} \right) \hfill \\ \end{gathered} \right] $$

where the first part of the formula (25) represents the kinetic molar specific heat (equation-26) and this part is independent of the absolute temperature. Besides, the second part represents the molar specific heat of the Morse interaction (equation-27), and as we see, the Morse part is depends on the absolute temperature and this part is proportional to inverse of the square of the absolute temperature which means that the Morse part is more effective in low temperature range. In addition, we see that the Morse part of the molar specific heat is depends on the well depth of Morse potential and is proportional to the square of the well depth. Also, the Morse part of the Molar specific heat depends on the diameter of the particles composing the described system, and the parameter which determine the width of the Morse potential, and the bond distance, and the volumetric density of the system.

### Applications of the Morse specific heat formula

We use the formula of the Morse specific heat which we derived for some application where we start from the hydrogen fluoride (HF) where we calculated the specific heat at the room temperature and we illustrated the result of this application in Table 1 which contains the molar mass of the hydrogen fluoride molecule, the density (volume) of the hydrogen fluoride, and the specific heat of the hydrogen fluoride molecule.

**Table 1:** The specific heat of the hydroen fluoride at the room temperature

*t(C*^*O*^*)*	*M (g/mol)*	*ρ (g/cc)*	*C*^*V*^* (J/gK)*
25	20.0100	0.00115	0.6230

Besides, we use the formula for finding the specific heat of the hydrogen chloride (HCl) at the temperature and we illustrated the result in the Table 2 which contains the molar mass of the hydrogen chloride molecule, the density (volume) of the hydrogen chloride, and the specific heat of the hydrogen chloride molecule.

**Table 2:** The specific heat of the hydrogen chloride at the room temperature.

*t(C*^*O*^*)*	*M (g/mol)*	*ρ(g/cc)*	*C*^*V*^_*M*_*(J/gK)*
25	36.46	0.00149	0.3419

The two previous applications of the formula which we derived are about the diatomic and linear molecules and are composed of two type of atoms, in addition, we use the formula of the specific heat of the Morse potential part specific heat for another part of the molecules which is Cs2 [23] (Caesium dimer) which is composed of one type of atoms. We calculated the Morse part specific heat for this dimer and the result of this calculation contained in Table 3 which includes the molar mass and the specific heat of this dimer.

**Table 3:** The specific heat of the caesium dimer at the room temperature.

*t(C*^*O*^*)*	*M (g/mol)*	$C_{M}^{V} (J/gK) \times 10^{ - 23}$
25	265.8109	9.2434

The result of the specific heat of the caesium dimer can be compared of the value resulted from the study [23] where we see that the two vibrational part of the specific heat are near to each other. Also, we use the formula of the specific heat of Morse potential for the lithium dimer for finding the vibrational part of the specific heat of this dimer at the room temperature and we illustrated the result of this calculation in Table 4 which includes the molar mass and the specific heat of this dimer.

**Table 4:** The specific heat of the lithium dimer at the room temperature.

*t(C*^*O*^*)*	*M (g/mol)*	$C_{M}^{V} (J/gK) \times 10^{ - 23}$
25	13.8800	3.4208

**Table 5:** The specific heat of the hydrogen and nitrogen at the room temperature.

	*t (C*^*O*^*)*	*M (g/mol)*	*ρ (g/cc)*	*C*^*V*^_*M*_* (J/gK)*
Hydrogen	25	2.0160	0.0009	6.1834
Nitrogen	25	28.0140	0.0013	0.4450

Finally, we use the formula of the specific heat for finding the specific heat at the room temperature of the hydrogen [25] and nitrogen [24] molecules.

As we see from the last table (Table 5), the specific heat of the hydrogen molecule and nitrogen molecule at the room temperature have the same rank of the values found in the literatures.

### The ratio *γ*

Additional significant property of the material which is the ratio *γ* can be found from the formula which we derived in this work where this property represents the ratio between the heat capacity at constant *V* and at constant *P* and defined as:

28 $$ \gamma = \frac{{C^{p} (T)}}{{C^{V} (T)}} $$

which becomes given by the following formula using the two equations-21 and 23:

29 $$ \gamma = 1 + \frac{{V\alpha^{2} \chi (T)T}}{{\left[ \begin{gathered} \frac{{3k_{B} N}}{2} + \frac{{2n\pi {\rm K}_{0}^{2} N}}{{k_{B} T^{2} }}\left( {2\exp \left( {2a\left( {r_{0} - d} \right)} \right)} \right)\left( {\frac{{d^{2} }}{a} + \frac{d}{{a^{2} }} + \frac{1}{{2a^{3} }}} \right) \hfill \\ + \frac{{\exp \left( {4a\left( {r_{0} - d} \right)} \right)}}{4}\left( {\frac{{d^{2} }}{a} + \frac{d}{{2a^{2} }} + \frac{1}{{8a^{3} }}} \right) - \frac{4}{3}\exp \left( {3a\left( {r_{0} - d} \right)} \right)\left( {\frac{{d^{2} }}{a} + \frac{2d}{{3a^{2} }} + \frac{2}{{9a^{3} }}} \right) \hfill \\ \end{gathered} \right]}}  $$

As we see from the ratio γ of the Morse potential the ratio depends on the Morse parameters and absolute temperature of the system in addition to the Bulk modulus of the system at this temperature.

## Conclusions

We derived a formula of the Morse potential specific heat using the theory of the integral equations. We employed the mean-spherical-approximation for that purpose. First, we found a formula for the total energy of the composition described with Morse interaction, and based on the energy formula, we derived the required formula of the molar specific heat for the Morse interaction. We derived two formulas of the heat capacity for the Morse interaction, one for the constant pressure and the other for the constant volume.

We found that, the molar specific heat capacity of the Morse potential is depends on the absolute temperature of the systems via the inverse-square low of the absolute temperature. Also, we found that the specific molar heat of the Morse interaction is function to the particles’ diameter, the bond distance of the Morse interaction, the width of the well parameter, the volumetric density of the system, and the depth of the Morse well. We found that the Morse molar specific heat is proportional to the square of the depth well of the Morse interaction. We applied the formula for six different molecules which are the lithium and caesium dimers, the hydrogen fluoride, hydrogen chloride, nitrogen, and hydrogen molecules and we found that the values are near the values in other literatures.

The derived formula in the present work is applied for finding the specific heat capacity for the oscillations part as a general case which is represented via the Morse potential, for instance, the diatomic molecules as the hydrogen chloride molecule and hydrogen fluoride molecule.

## Data Availability

All data generated or analysed during this study are included in this published article.
